# Supplementary material for: Therapeutic Efficacy of Curcumin Enhanced by Microscale Discoidal Polymeric Particles in a Murine Asthma Model
Source: Pharmaceutics. 2020 Aug 6;12(8):739. doi: 10.3390/pharmaceutics12080739 (PMC7463530; doi:10.3390/pharmaceutics12080739)
Supplement: Supplementary file 1 [file pharmaceutics-12-00739-s001.pdf]

pharmaceutics

Supplementary Materials: Enhanced therapeutic efficacy of curcumin by microscale discoidal polymeric particles in a murine asthma model

Jun Young Park, Ga Eul Chu, Sanghyo Park, Chaewon Park, Susmita Aryal, Won Jun Kang, Won Gil Cho and Jaehong Key

Table S1. Effect of Cur-PLGA-DPPs administration on hematological parameters. Shown are mean values ± standard deviation (n = 4).

Parameter	Control	OVA	OVA + Cur-PLGA-DPPs 5 mg/kg	OVA + Cur-PLGA-DPPs 25 mg/kg	OVA + Curcumin 4 mg/kg
Red blood cell (M/μL)	11.2 ± 1.2	14.1 ± 1.7	10.9 ± 0.7	11.4 ± 0.7	14.2 ± 1.1
Hemoglobin (g/dL)	18.7 ± 2.0	21.8 ± 1.2	17.1 ± 1.2	16.5 ± 0.9	23.0 ± 2.2
Hematocrit (%)	66.7 ± 6.4	82.2 ± 6.4	62.2 ± 3.7	59.7 ± 3.0	82.6 ± 8.0
Mean corpuscular volume (fL)	59.9 ± 1.4	58.6 ± 2.6	57.1 ± 1.2	52.4 ± 1.4	58.2 ± 1.1
Mean Cell Hemoglobin (MCH; pg)	16.8 ± 0.5	15.6 ± 1.0	15.6 ± 0.5	14.5 ± 0.6	16.2 ± 0.6
MCH Concentration (g/dL)	28.0 ± 0.7	26.6 ± 1.2	27.4 ± 0.4	27.7 ± 0.4	27.8 ± 0.8
White blood cell (k/μL)	4.9 ± 3.6	4.5 ± 1.2	3.1 ± 0.3	6.6 ± 1.2	6.8 ± 1.1
Neutrophil (k/μL)	0.6 ± 0.4	0.8 ± 0.3	0.7 ± 0.1	1.4 ± 0.2	1.6 ± 0.2
Lymphocyte (k/μL)	4.0 ± 3.1	3.2 ± 1.0	2.9 ± 1.7	4.8 ± 1.0	4.9 ± 0.9
Monocyte (k/μL)	0.2 ± 0.2	0.3 ± 0.1	0.3 ± 0.1	0.4 ± 0.1	0.3 ± 0.0
Eosinophil (k/μL)	0.0 ± 0.0	0.1 ± 0.0	0.0 ± 0.0	0.0 ± 0.0	0.1 ± 0.0
Basophil (k/μL)	0.0 ± 0.0	0.0 ± 0.0	0.0 ± 0.0	0.0 ± 0.0	0.0 ± 0.0
Platelet (k/μL)	853.0 ± 38.7	979.5 ± 200.1	651.7 ± 64.3	607.0 ± 31.5	835.0 ± 86.2
